# Biofilm Formation, Virulence-Associated Genes, and Antimicrobial Resistance in *Proteus mirabilis* Isolates from Urinary Tract Infections in Iran

**DOI:** 10.3390/microorganisms14061242

**Published:** 2026-05-31

**Authors:** Mehdi Choori, Fateh Rahimi, Ali Qasemi, Mohammad Katouli

**Affiliations:** 1 Department of Cell and Molecular Biology & Microbiology, Faculty of Biological Science and Technology, University of Isfahan, Isfahan 81746-73441, Iran; mehdi.choori@gmail.com (M.C.); f.rahimi@sci.ui.ac.ir (F.R.); 2 Department of Biology, Faculty of Science, University of Sistan and Baluchestan, Zahedan 98167-45845, Iran; qasemi@science.usb.ac.ir; 3 Centre for Bioinnovation and School of Science, Technology and Engineering, University of the Sunshine Coast, Maroochydore, QLD 4556, Australia

**Keywords:** *Proteus mirabilis*, urinary tract infection, biofilm formation, antimicrobial resistance, ERIC-PCR, virulence factors

## Abstract

*Proteus mirabilis* is a frequent cause of complicated urinary tract infections (UTIs), in which biofilm formation and antimicrobial resistance contribute to persistence and therapeutic failure. This study investigated the biofilm-forming capacity, antimicrobial resistance profiles, virulence-associated genes, and genetic diversity of *P. mirabilis* isolates recovered from UTI patients in Isfahan, Iran. A total of 104 non-duplicate clinical isolates were analyzed. Biofilm formation was quantified using a microtiter plate crystal violet assay, and antimicrobial susceptibility testing was performed according to CLSI guidelines. Extended-spectrum β-lactamase (ESBL) production and extended-spectrum cephalosporin resistance (ESCR) were assessed phenotypically and by PCR. Selected virulence- and biofilm-associated genes were detected by PCR, and clonal relatedness was evaluated using ERIC-PCR. Most isolates were capable of biofilm formation, with 51% classified as strong and 45.2% as moderate producers. High carriage rates of virulence- and biofilm-associated genes, including *zap*A, *zap*D, *ure*C, *ure*R, *lux*S, *rsb*A, and *acr*A, were observed. ESBL production and ESCR phenotypes were detected in 6.7% and 12.5% of isolates, respectively, while multidrug resistance was observed in 30.4% of isolates. ERIC-PCR analysis identified predominant clonal clusters among isolates exhibiting strong biofilm production. These findings highlight the coexistence of biofilm formation, virulence determinants, antimicrobial resistance, and clonal diversity in uropathogenic *P. mirabilis* in this region.

## 1. Introduction

Urinary tract infections (UTIs) are among the most prevalent bacterial infections worldwide. They affect an estimated 150 million individuals annually and impose a substantial burden on healthcare systems and economies. Recurrent UTIs are particularly common among women, with lifetime prevalence estimates ranging from 40% to 60% [1]. Although *Escherichia coli* remains the leading cause of UTIs, other pathogens, such as *Proteus mirabilis*, *Klebsiella pneumoniae*, and *Pseudomonas aeruginosa*, also play significant roles in both uncomplicated and complicated infections [2].

The global rise in antimicrobial resistance (AMR) has markedly complicated the clinical management of UTIs. Increasing resistance to commonly prescribed first and second-line antimicrobial agents has been widely documented. This limits empirical treatment options and increases the risk of therapeutic failure [3]. This challenge is particularly pronounced in complicated UTIs, where MDR organisms are frequently encountered, and antimicrobial susceptibility testing becomes essential for guiding effective therapy [4].

Among non-*E. coli* uropathogens, *P. mirabilis* is strongly associated with complicated UTIs, especially in patients with long-term urinary catheterization [5]. A defining feature of this organism is its potent urease activity. This enzyme hydrolyzes urea to ammonia, elevates urinary pH, and promotes the precipitation of calcium and magnesium salts. This process leads to catheter encrustation, stone formation, and obstruction, facilitating catheter-associated UTIs (CA-UTIs) and persistent infections [5]. In addition to CA-UTIs, *P. mirabilis* infections may progress to pyelonephritis, bacteremia, and sepsis, underscoring their clinical significance.

The pathogenicity of *P. mirabilis* is mediated by various virulence-associated factors. These include hemolysin, Proteus toxic agglutinin, fimbrial adhesins, flagella, urease, and biofilm formation [6,7]. Biofilm formation plays a central role in the persistence of *P. mirabilis* infections. It enables bacterial adherence to biotic and abiotic surfaces, such as uroepithelial cells and urinary catheters, and by conferring protection against host immune defenses and antimicrobial agents [8,9]. Several virulence-associated genes, including those related to swarming motility, fimbriae, urease regulation, quorum sensing, and efflux systems. These genes may also contribute indirectly to antimicrobial resistance phenotypes [10].

Advances in whole-genome sequencing (WGS) have substantially expanded current knowledge of *P. mirabilis* virulence. This includes identifying complex regulatory networks such as two-component systems and other accessory genes involved in environmental adaptation and pathogenicity [11,12]. However, routine implementation of WGS remains limited in many clinical and regional surveillance settings. Consequently, phenotypic characterization combined with targeted molecular detection of selected virulence- and biofilm-associated genes, remains crucial for understanding the epidemiology and pathogenic potential of *P. mirabilis* isolates in real-world clinical contexts [9,13]. Previous studies have reported considerable heterogeneity in biofilm-forming capacity, virulence gene carriage, and antimicrobial resistance profiles among *P. mirabilis* strains from different geographical regions [14]. While such studies provide valuable descriptive insights, regional data are often unevenly distributed. Additionally, findings from one setting may not be directly generalizable to another due to differences in patient populations, antimicrobial usage patterns, and circulating clonal lineages.

In Iran, UTIs represent a significant public health concern; however, comprehensive data on the genetic diversity, biofilm-forming capacity, and resistance characteristics of *P. mirabilis* isolates remain limited. Although global studies have elucidated many aspects of *P. mirabilis* pathogenicity, region-specific surveillance is necessary to define local strain characteristics better. This will support evidence-based antimicrobial stewardship [15,16].

Therefore, the present study aimed to provide a descriptive characterization of biofilm-producing *P. mirabilis* isolates recovered from UTI patients in Iran. Specifically, we evaluated biofilm-forming capacity, antimicrobial susceptibility patterns, and the distribution of selected virulence- and biofilm-associated genes. Additionally, we assessed genetic relatedness using ERIC-PCR. By integrating phenotypic and targeted molecular data, this study contributes region-specific epidemiological information and supports ongoing efforts to improve the management of *P. mirabilis*-associated UTIs.

## 2. Materials and Methods

### 2.1. Bacterial Isolates

Between May 2021 and December 2022, a total of 104 non-duplicate *P. mirabilis* clinical isolates were recovered from urine samples of hospitalized patients at Al-Zahra University Hospital, Isfahan, Iran. The isolates were obtained as part of routine microbiological diagnostics and represented a heterogeneous patient population. The isolates originated from 42 male patients and 62 female patients. The age range for male patients was 7 months to over 90 years (median age: 70 years), while that for female patients was 12 months to over 90 years (median age: 52 years). This study was approved by the Ethics Committee of the University of Isfahan (Approval No. IR.UI.REC.1401.126). All specimens consisted of residual, de-identified clinical samples collected by the hospital microbiology laboratory during standard patient care. The authors had no involvement in patient recruitment, clinical management, or sample collection. Informed consent was obtained in accordance with the hospital’s internal regulations. This included consent from parents or legal guardians for minor patients. Primary isolation was performed on MacConkey agar (Merck, Darmstadt, Germany). Lactose-non-fermenting colonies with characteristic morphology were subjected to standard biochemical identification procedures, as previously described [17]. Molecular confirmation of *P. mirabilis* was performed by polymerase chain reaction (PCR) targeting the species-specific *ure*C and *ure*R genes using published primers [18] (Table 1). Confirmed isolates were preserved in tryptic soy broth (TSB; Merck, Darmstadt, Germany) supplemented with 25% glycerol. They were stored at −20 °C until further phenotypic and molecular analyses were performed.

### 2.2. Biofilm Formation Assay

Biofilm formation was quantified using a standard crystal violet (CV) microtiter plate assay to assess adherence to abiotic surfaces, as previously described [29]. Briefly, overnight bacterial cultures grown in Luria–Bertani (LB) broth were adjusted to approximately 1 × 10^8^ CFU/mL. These cultures were subsequently diluted 1:100 in fresh LB broth. Aliquots of 200 µL of the diluted suspensions were inoculated into sterile 96-well flat-bottom polystyrene microplates (Greiner Bio-One, Monroe, NC, USA) in triplicate for each isolate. Sterile LB broth without bacterial inoculation served as a negative control. Plates were incubated at 37 °C for 24 h under static conditions to allow biofilm development. Following incubation, the contents of the wells were gently aspirated. The wells were then washed three times with phosphate-buffered saline (PBS; pH 7.4) to remove planktonic and loosely attached cells. Plates were then air-dried at room temperature. Adherent biofilms were stained with 0.3% (*w*/*v*) crystal violet solution (Sigma-Aldrich, Taufkirchen, Germany) for 15 min. Excess stain was removed by rinsing the wells thoroughly with distilled water. The bound crystal violet was solubilized using an acetone–ethanol mixture (20:80, *v*/*v*). The optical density (OD) was measured at 570 nm using a multimode microplate reader (Infinite™ M200 PRO, Tecan, Männedorf, Switzerland). The optical density cut-off value (ODc) was defined as the mean OD of the negative control plus three standard deviations. Based on OD measurements, isolates were categorized as weak (ODc < OD ≤ 2ODc), moderate (2ODc < OD ≤ 4ODc), or strong (OD > 4ODc) biofilm producers, following the criteria described by Aniba et al. [30]. The biofilm formation assay was performed with three independent repeats on different days. For positive control, we used *P. aeruginosa* PTCC 1707, a well-characterized strain known for its strong biofilm-forming ability. This approach ensures the reliability and validity of our biofilm assay results.

### 2.3. Antibiotic Susceptibility Testing

Antimicrobial susceptibility testing was performed using the Kirby–Bauer disk diffusion method on Mueller–Hinton agar (MHA; Scharlau, Sentmenat, Spain) in accordance with the Clinical and Laboratory Standards Institute (CLSI) guidelines [31]. Briefly, bacterial suspensions equivalent to a 0.5 McFarland standard were prepared in sterile normal saline. These suspensions were then evenly inoculated onto the surface of MHA plates using sterile swabs. The following antibiotic disks (Liofilchem, Roseto degli Abruzzi, Italy) were applied: ampicillin (10 µg), amoxicillin/clavulanic acid (20/10 µg), piperacillin/tazobactam (100/10 µg), cefotaxime (30 µg), ceftazidime (30 µg), ceftriaxone (30 µg), cefepime (30 µg), cefoxitin (30 µg), imipenem (10 µg), meropenem (10 µg), gentamicin (10 µg), kanamycin (30 µg), amikacin (30 µg), ciprofloxacin (5 µg), levofloxacin (5 µg), ofloxacin (5 µg), and trimethoprim/sulfamethoxazole (1.25/23.75 µg). Plates were incubated aerobically at 37 °C for 18–24 h. Inhibition zone diameters were then measured and interpreted as susceptible, intermediate, or resistant according to CLSI interpretive criteria [31]. Multidrug resistance (MDR) was defined as non-susceptibility to at least one antimicrobial agent in three or more antimicrobial classes, in accordance with internationally accepted consensus definitions.

### 2.4. Phenotypic Screening of ESBL and ESCR

Phenotypic detection of extended-spectrum β-lactamase (ESBL) production was performed using the combined disk diffusion method, in accordance with the Clinical and Laboratory Standards Institute (CLSI) recommendations [32]. Briefly, disks containing ceftazidime (30 µg) and cefotaxime (30 µg), alone and in combination with clavulanic acid (10 µg), were placed on Mueller-Hinton agar plates. The plates had been previously inoculated with standardized bacterial suspensions (0.5 McFarland). After incubation at 37 °C for 18–24 h, the inhibition zone diameters were analyzed. An increase of ≥5 mm in the zone diameter for either ceftazidime or cefotaxime in the presence of clavulanic acid, compared with the corresponding disk without clavulanic acid, was interpreted as indicative of ESBL production. Extended-spectrum cephalosporin resistance (ESCR) was defined phenotypically based on resistance to at least one third-generation cephalosporin, including cefotaxime, ceftazidime, or ceftriaxone, according to CLSI interpretive criteria. *E. coli* ATCC 25922 and *K. pneumoniae* ATCC 700603 were used as ESBL-negative and ESBL-positive quality control strains, respectively, to ensure the accuracy and reliability of the phenotypic assays [20].

### 2.5. Detection of Virulence and Biofilm-Associated Genes

Genomic DNA was extracted from overnight bacterial cultures using a simple boiling method, as previously described [20]. Briefly, bacterial cells were suspended in sterile distilled water and heated at 100 °C for 10 min. Subsequently, the suspension was centrifuged to remove cellular debris. The resulting supernatant containing crude DNA was used as the template for PCR amplification. The presence of selected biofilm-associated [33] and virulence-related genes was investigated by conventional PCR using gene-specific primers (Table 1). The targeted genes were chosen based on their reported involvement in adhesion, biofilm formation, urease activity, quorum sensing, and motility in *P. mirabilis*. PCR amplification was carried out under the following conditions: initial denaturation step at 95 °C for 5 min; 30 cycles of denaturation at 95 °C for 30 s, annealing at gene-specific temperatures (Table S1) for 30 s, and extension at 72 °C for 30 s; followed by a final extension at 72 °C for 5 min. Amplified products were separated by agarose gel electrophoresis. They were then stained with an appropriate nucleic acid stain and visualized under ultraviolet illumination.

#### Detection of β-Lactamase Genes

The presence of β-lactamase-encoding genes of all bacterial isolates was examined using multiplex PCR assays targeting the most prevalent ESBL gene families, as described previously [34]. These assays were designed to detect commonly reported β-lactamase genes associated with ESCR. Primer sequences and expected amplicon sizes are listed in Table 1. For clarity, β-lactamase gene families are presented without the “*bla*” prefix in the primer tables and Methods in Section 2. However, the prefix “*bla*” is used in the Results and Discussion (Section 3 and Section 4) to denote β-lactamase-encoding genes. PCRs were performed under conditions optimized for multiplex amplification. PCR products were analyzed by electrophoresis on a 1% agarose gel as described above.

### 2.6. ERIC-PCR Genotyping

Genetic relatedness among the biofilm-producing *P. mirabilis* isolates was assessed using enterobacterial repetitive intergenic consensus polymerase chain reaction (ERIC-PCR), as previously described [26]. ERIC-PCR was employed as a fingerprinting method to evaluate genetic diversity and to identify predominant clonal patterns within the isolate collection. Amplified ERIC-PCR products were separated by agarose gel electrophoresis. Banding patterns were analyzed using GelQuest software (version 2.0). Similarity coefficients were calculated based on band presence or absence. Clustering analysis was performed using standard similarity algorithms. Isolates exhibiting ≥ 90% similarity were considered clonally related and assigned to the same ERIC type. In contrast, those with similarity values below this threshold were classified as distinct genotypes [35].

### 2.7. Statistical Analysis

Statistical analyses were performed using SPSS software (version 24). The Chi-square test was used to compare the overall distribution of isolates by sex (Table 2). For the assessment of associations between biofilm-forming capacity and antimicrobial resistance profiles (Table 3), Fisher’s exact test (Freeman–Halton extension) was applied, as this method is more appropriate for categorical data with small expected cell counts. Due to the very small sample size in the weak biofilm group (n = 4), statistical analysis for the distribution of virulence- and biofilm-associated genes (Table 4) was not considered reliable; therefore, results are presented descriptively. All statistical tests were two-tailed, and a *p*-value of <0.05 was considered statistically significant.

## 3. Results

### 3.1. Distribution of Isolates and Biofilm Formation

**Figure 1:**
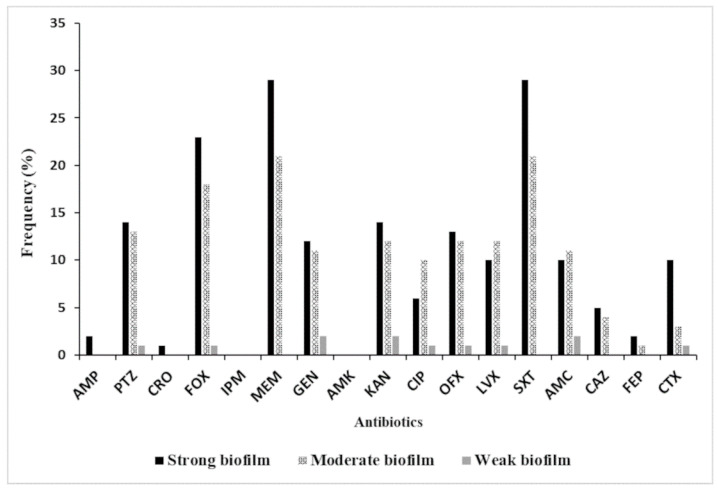
Association between biofilm-forming capacity and antimicrobial resistance among *P. mirabilis* isolates recovered from urinary tract infections. Antibiotics tested include ampicillin (AMP), piperacillin/tazobactam (PTZ), ceftriaxone (CRO), cefoxitin (FOX), imipenem (IPM), meropenem (MEM), gentamicin (GEN), amikacin (AMK), kanamycin (KAN), ciprofloxacin (CIP), ofloxacin (OFX), levofloxacin (LVX), trimethoprim/sulfamethoxazole (SXT), amoxicillin/clavulanic acid (AMC), ceftazidime (CAZ), cefepime (FEP), and cefotaxime (CTX).

Overall, *P. mirabilis* isolates were recovered more frequently from female patients (59.6%, n = 62) than from male patients (40.5%, n = 42), and this difference was statistically significant (*p* = 0.0071). When stratified by age, however, a higher isolation frequency was observed among elderly male patients (>70 years) compared with females in the same age group, as shown in Table 2. Assessment of biofilm formation revealed that the majority of isolates were capable of forming biofilms under the tested conditions. Specifically, 53 isolates (50.96%) were classified as strong biofilm producers, 47 isolates (45.2%) as moderate biofilm producers. Only 4 isolates (3.8%) were weak biofilm producers Figure 1. These findings indicate a marked predominance of moderate-to-strong biofilm-forming phenotypes among the clinical *P. mirabilis* isolates examined in this study.

### 3.2. Antibiotic Susceptibility Patterns

Antimicrobial susceptibility testing showed that all isolates were susceptible to piperacillin/tazobactam, cefoxitin, and meropenem. In contrast, resistance to at least one tested antibiotic was observed in 63 isolates (60.6%). Based on the applied definition, 32 isolates (30.8%) were classified as MDR, exhibiting non-susceptibility to at least one agent in three or more antimicrobial categories. The distribution of resistance frequencies across biofilm-forming groups is presented in Table 3. Among strong biofilm producers (n = 53), the highest resistance rates were observed for trimethoprim/sulfamethoxazole (58.49%), kanamycin (41.51%), ampicillin (28.3%), gentamicin (26.41%), ciprofloxacin (24.52%), and levofloxacin (24.52%). Among moderate biofilm producers (n = 47), resistance was most frequently observed for trimethoprim/sulfamethoxazole (46.81%), kanamycin (42.55%), ofloxacin (27.7%), and gentamicin (25.5%). In the weak biofilm group (n = 4), resistance rates were generally lower, though this group was too small for meaningful comparison. Fisher’s exact test (Freeman–Halton extension) revealed no statistically significant association between biofilm-forming capacity and resistance to any of the tested antimicrobial agents (*p* > 0.05 for all comparisons, Table 3). These findings indicate that while descriptive differences in resistance frequencies were observed across biofilm-forming groups, these differences did not reach statistical significance, likely reflecting the limited size of the weak biofilm group and the overall distribution of resistance in this collection. Phenotypic screening identified seven isolates (6.7%) as ESBL producers, based on a ≥5 mm increase in inhibition zone diameter in the presence of clavulanic acid. In addition, the ESCR phenotype was detected in 13 isolates (12.5%), indicating reduced susceptibility to third-generation cephalosporins among a subset of the studied strains.

### 3.3. Distribution of Virulence- and Biofilm-Associated Genes

PCR analysis demonstrated a high prevalence of virulence- and biofilm-associated genes among the *P. mirabilis* isolates. The *zap*A, *zap*D, and *rsb*A genes were detected in all isolates except one, whereas the *hly*A gene was not identified in any isolate. In addition, all strains were uniformly positive for *ure*C, *ure*R, *lux*S, and *acr*A, indicating the widespread distribution of genes associated with urease activity, quorum sensing, and efflux-related mechanisms among the isolates. The overall distribution of virulence-associated genes is summarized in Table 4. Due to the very small sample size in the weak biofilm group (n = 4), formal statistical comparisons were not considered reliable; therefore, results are presented descriptively. For most genes tested, the prevalence was similarly high across all biofilm-forming groups, suggesting a largely conserved virulence gene repertoire regardless of biofilm-forming capacity. Notably, minor variations in the prevalence of *uca* and *acr*B were observed across groups; however, no statistically significant association was observed between the presence of any virulence-associated gene and biofilm-forming capacity. These findings suggest that the virulence gene repertoire of *P. mirabilis* is largely independent of biofilm-forming capacity in this collection. However, larger studies with more balanced group sizes would be needed to confirm this observation.

### 3.4. β-Lactamase Gene Profiles

**Figure 2:**
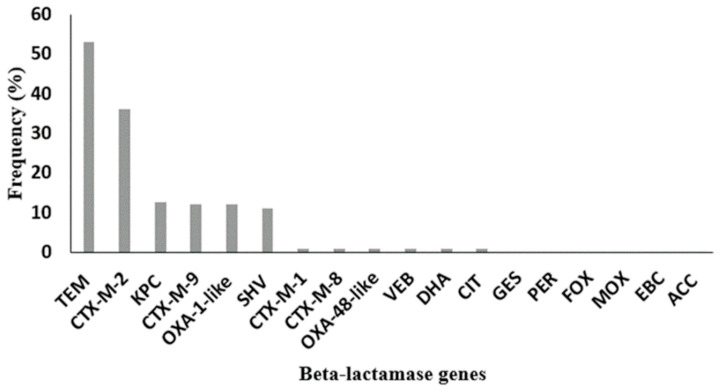
Frequency distribution of β-lactamase gene families detected among biofilm-producing *P. mirabilis* isolates. Gene families are indicated according to the corresponding β-lactamase types identified by PCR.

Molecular screening for β-lactamase-encoding genes showed that *bla*TEM (52.9%) and *bla*CTX-M-2 (36.5%) were the most frequently detected genes, followed by *bla*KPC (12.5%), *bla*CTX-M-9 (11.5%), *bla*OXA-1-like (11.5%), and *bla*SHV (10.6%) (Figure 2). Overall, 78.8% of isolates carried at least one β-lactamase gene, indicating a substantial genetic basis for β-lactam resistance among the studied strains. The distribution of β-lactamase genes differed across biofilm-forming categories, with higher carriage rates generally observed among strong biofilm-producing isolates. However, due to the limited number of weak biofilm producers, no formal statistical comparison was performed between biofilm categories for individual resistance genes.

### 3.5. Genotypic Diversity of Isolates

**Figure 3:**
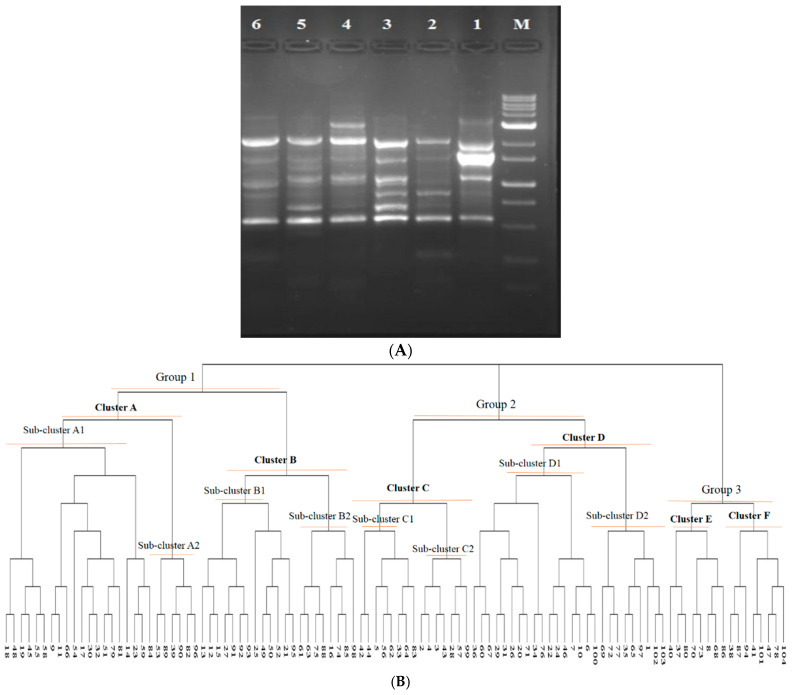
ERIC-PCR genotyping of biofilm-producing *P. mirabilis* isolates: (**A**) Representative ERIC-PCR banding patterns of selected *P. mirabilis* isolates obtained by agarose gel electrophoresis. Lane M indicates a 100 bp DNA ladder used as the molecular size marker. (**B**) Dendrogram showing the genetic relatedness of isolates based on ERIC-PCR profiles, generated using GelQuest software (version 2.0) at a ≥90% similarity threshold, resulting in six major clusters (A–F).

ERIC-PCR fingerprinting revealed substantial genotypic heterogeneity among the biofilm-producing *P. mirabilis* isolates. Representative banding patterns obtained by 1% agarose gel electrophoresis are shown in Figure 3A, and the corresponding dendrogram based on similarity analysis is presented in Figure 3B. Using GelQuest software (version 2.0), a total of 63 distinct ERIC-PCR profiles were identified, comprising 40 common types (CTs; shared by two or more isolates) and 23 single types (STs). Cluster analysis performed at a ≥90% similarity threshold grouped the isolates into six major clusters (A–F). Clusters A and D were the most prevalent, each accounting for approximately 25% of the isolates, whereas clusters E and F were the least frequent, each representing 8% of the total collection. Strong biofilm-producing isolates were disproportionately represented within clusters E (75%) and A (73.1%), as shown in Table 5. Differences in antimicrobial resistance profiles were also observed among clusters. Isolates belonging to cluster D exhibited the highest resistance rate to ampicillin (46.2%), while cluster E showed the highest frequency of resistance to trimethoprim/sulfamethoxazole (75%), as shown in Table 6. Analysis of virulence- and biofilm-associated genes demonstrated largely conserved gene carriage across clusters. Specifically, with *zap*A detected uniformly among all genotypes. In contrast, *zap*D and *rsb*A showed variable distributions, particularly within clusters A and B. The *hmp*A gene was detected across most clusters, with the highest prevalence observed in clusters B and D. In addition, *mrp*A and *uca* were detected in all isolates belonging to cluster E. The *mrp*H was most frequently observed in clusters A and D, as shown in Table 7. These findings indicate cluster-specific patterns of virulence gene distribution without implying direct functional or mechanistic associations.

## 4. Discussion

Biofilm formation is a well-recognized contributor to the pathogenicity of *P. mirabilis*, particularly in CA-UTIs and other complicated urinary tract infections. In the present study, more than half of the isolates were classified as strong biofilm producers. This finding is consistent with previous reports from Iran [9,36] but indicates a higher frequency compared to studies from Turkey and Brazil, where strong biofilm production ranged between 30% and 40% [13,35]. Such geographic variation may reflect differences in circulating clonal lineages, patient demographics, antibiotic exposure, and local infection control practices.

Biofilm formation substantially enhances bacterial persistence by shielding embedded cells from host immune defenses and reducing antibiotic penetration [37]. In the present study, higher resistance frequencies were descriptively observed among strong and moderate biofilm producers for several antimicrobial agents, including trimethoprim/sulfamethoxazole and aminoglycosides. However, Fisher’s exact test did not reveal any statistically significant association between biofilm-forming capacity and resistance to the tested antimicrobial agents (*p* > 0.05 for all comparisons). These findings should be interpreted cautiously, as the small size of the weak biofilm group (n = 4) may have limited the statistical power to detect potential differences. These observations nonetheless highlight the clinical importance of biofilm-associated *P. mirabilis* infections and support continued investigation of alternative therapeutic strategies, including biofilm-disrupting agents, anti-biofilm catheter coatings, and quorum-sensing inhibitors [38,39].

Antimicrobial resistance among *P. mirabilis* isolates represents a growing global concern in UTI management [40]. In this study, 30.37% of isolates were classified as MDR, with high resistance rates observed for trimethoprim–sulfamethoxazole, kanamycin, and ampicillin. Similar resistance patterns have been reported in studies from India and Pakistan [41,42]. The widespread empirical use of trimethoprim-sulfamethoxazole as a first-line therapy for UTIs likely contributes to elevated resistance rates in many regions [35,43]. In contrast, the preserved susceptibility of isolates to piperacillin-tazobactam, cefoxitin, and meropenem suggests that these agents remain effective therapeutic options for severe *P. mirabilis* infections in Iran, consistent with reports from Europe and the United States [44,45]. The absence of certain β-lactamase genes, such as *bla*GES and *bla*PER, further highlights regional differences in resistance mechanisms and emphasizes the importance of local surveillance data to guide empirical therapy [46].

The detection of ESBL-producing (6%) and ESCR (13%) isolates in this study aligns with reports from Saudi Arabia and Egypt, indicating a moderate but clinically relevant burden of β-lactam resistance in the region [8,47,48,49]. These findings reinforce the need for routine screening for β-lactamase production and for strict antimicrobial stewardship to limit the further dissemination of resistant strains. The observed discrepancy between the high prevalence of β-lactamase-encoding genes and the relatively low rate of phenotypic ESBL production warrants consideration. While *bla*TEM (52.9%) and *bla*CTX-M-2 (36.5%) were the most frequently detected genes, only 6.7% of isolates were phenotypically confirmed as ESBL producers. This genotype–phenotype discordance may be attributed to several factors. First, certain β-lactamase variants, such as *bla*TEM-1, encode narrow-spectrum enzymes that do not confer ESBL activity and are therefore not detected by the combined disk diffusion method. Second, the presence of a resistance gene does not guarantee its transcriptional expression, as gene silencing or regulatory suppression may limit phenotypic manifestation. Third, enzymes such as KPC-type carbapenemases are not reliably detected by standard ESBL phenotypic methods. Such genotype–phenotype discordance has been previously reported in Enterobacteriaceae [46].

Virulence-associated genes are closely intertwined with biofilm formation and antimicrobial resistance in *P. mirabilis*. In this study, key virulence-associated genes, including *ure*C, *ure*R, *zap*A, and *zap*D, were highly prevalent, consistent with previous reports from Iran [15] and Egypt [13,49]. These genes are involved in urease activity, tissue damage, and biofilm maturation, all of which play critical roles in catheter encrustation and persistence in CA-UTIs [6,50]. The high prevalence of *zap*A and *zap*D among strong biofilm-producing isolates supports their potential relevance in biofilm-associated pathogenicity; however, functional relationships were not directly assessed in this study [51,52,53].

Genotyping by ERIC-PCR revealed substantial genetic diversity among the isolates, with 63 unique profiles grouped into six major clonal clusters. Notably, two dominant clusters were enriched with strong biofilm producers and exhibited higher levels of antimicrobial resistance, suggesting a potential selective advantage for these lineages in the clinical setting. Although ERIC-PCR has lower resolution than whole-genome-based typing methods, the observed clustering patterns are comparable to those reported in studies from China and Brazil, where certain biofilm-forming clonal groups appeared better adapted for persistence and dissemination in healthcare environments [43,54].

Although ERIC-PCR was employed to assess genotypic diversity among isolates, it is known to have limitations in reproducibility and discriminatory power compared to sequence-based approaches such as multilocus sequence typing (MLST) or WGS. In future studies, incorporating higher-resolution typing methods such as WGS or at least MLST would provide a more detailed understanding of the clonal relatedness among Proteus mirabilis isolates. These approaches could also reveal the genomic location of β-lactamase genes, thereby helping to explain the observed phenotype-genotype discrepancies and further strengthening the genetic analysis of antimicrobial resistance.

In the present study, no statistically significant associations were observed between biofilm-forming capacity, virulence gene carriage, and antimicrobial resistance profiles. These results should be interpreted in the context of the study’s limitations. The weak biofilm group comprised only four isolates, which substantially reduced statistical power and precluded reliable formal comparisons for this subgroup. Therefore, the absence of statistically significant associations in the current dataset does not necessarily indicate the absence of a biological relationship. Larger, prospective studies with more balanced group sizes are warranted to further investigate the potential interactions between biofilm formation, virulence gene expression, and antimicrobial resistance in *P. mirabilis*.

Overall, the coexistence of strong biofilm formation, multidrug resistance, and high carriage of virulence-associated genes among genetically diverse *P. mirabilis* isolates highlights the complexity of managing UTIs caused by this pathogen. While the descriptive nature of the study limits mechanistic interpretation, the findings underscore the importance of continuous regional surveillance, molecular epidemiology, and the development of biofilm-targeted interventions to improve infection control strategies and optimize treatment outcomes.

## 5. Conclusions

This study provides a descriptive overview of biofilm formation, antimicrobial resistance, virulence-associated gene distribution, and genetic relatedness among *P. mirabilis* isolates recovered from patients with urinary tract infections in Isfahan, Iran. A substantial proportion of isolates demonstrated strong biofilm-forming capacity and multidrug-resistant phenotypes, alongside a high prevalence of key virulence-associated genes. Genotyping by ERIC-PCR revealed considerable genetic heterogeneity, with multiple clonal groups contributing to the local biofilm-producing isolate population. The coexistence of biofilm formation, antimicrobial resistance, and virulence gene carriage across genetically diverse strains highlights the complexity of managing *P. mirabilis*-associated UTIs. It limits the effectiveness of uniform empirical treatment approaches. Notably, no statistically significant associations were observed between biofilm-forming capacity and antimicrobial resistance or virulence gene carriage, likely reflecting the limited size of certain subgroups; larger studies are therefore needed to clarify these relationships. Although the study is descriptive in nature and does not address underlying molecular mechanisms, the findings underscore the importance of continuous regional surveillance integrating phenotypic and targeted molecular data. Such information is essential to support antimicrobial stewardship efforts and to inform future studies aimed at elucidating functional relationships between biofilm formation, virulence, and resistance in *P. mirabilis*.

## Figures and Tables

**Table 1:** Primers used in this study.

Primer Name	Gene Encoded for	Sequence (5 > 3)	Amplicon Size (bp)	Reference
*ure*C	Urease	F: GTTATTCGTGATGGTATGGG	533	[19]
		R: ATAAAGGTGGTTACGCCAGA		
*ure*R		F: GGTGAGATTTGTATTAATGG	225	[18]
		R: ATAATCTGGAAGATGACGAG		
Multiplex I TEM, SHV, and OXA-1-like	TEM variants, including TEM-1 and TEM-2	CATTTCCGTGTCGCCCTTATTC	800	[20]
		CGTTCATCCATAGTTGCCTGAC		
	SHV variants, including SHV-1	AGCCGCTTGAGCAAATTAAAC	713	
		ATCCCGCAGATAAATCACCAC		
	OXA-1, OXA-4 and OXA-30	GGCACCAGATTCAACTTTCAAG	564	
		GACCCCAAGTTTCCTGTAAGTG		
Multiplex II CTX-M group 1, group 2, and group 9	variants of the CTX-M group 1, including CTX-M-1, CTX-M-3, and CTX-M-15	TTAGGAARTGTGCCGCTGYA	688	
		CGATATCGTTGGTGGTRCCAT		
	variants of the CTX-M group 2, including CTX-M-2	CGTTAACGGCACGATGAC	404	
		CGATATCGTTGGTGGTRCCAT		
	variants of the CTX-M group 9, including CTX-M-9 and CTX-M-14	TCAAGCCTGCCGATCTGGT	561	
		TGATTCTCGCCGCTGAAG		
CTX-M group 8/25	CTX-M-8, CTX-M-25, CTX-M-26 and CTX-M-39 to CTX-M-41	AACRCRCAGACGCTCTAC	326	
		TCGAGCCGGAASGTGTYAT		
Multiplex III ACC, FOX, MOX, DHA, CIT, and EBC	ACC-1 and ACC-2	CACCTCCAGCGACTTGTTAC	346	
		GTTAGCCAGCATCACGATCC		
	FOX-1 to FOX-5	CTACAGTGCGGGTGGTTT	162	
		CTATTTGCGGCCAGGTGA		
	MOX-1, MOX-2, CMY-1, CMY-8 to CMY-11 and CMY-19	GCAACAACGACAATCCATCCT	895	
		GGGATAGGCGTAACTCTCCCAA		
	DHA-1 and DHA-2	TGATGGCACAGCAGGATATTC	997	
		GCTTTGACTCTTTCGGTATTCG		
	LAT-1 to LAT-3, BIL-1, CMY-2 to CMY-7, CMY-12 to CMY-18 and CMY-21 to CMY-23	CGAAGAGGCAATGACCAGAC	538	
		ACGGACAGGGTTAGGATAGY		
	ACT-1 and MIR-1	CGGTAAAGCCGATGTTGCG	683	
		AGCCTAACCCCTGATACA		
Multiplex IV VEB, PER, and GES	GES-1 to GES-9 and GES-11	AGTCGGCTAGACCGGAAAG	399	
		TTTGTCCGTGCTCAGGAT		
	PER-1 and PER-3	GCTCCGATAATGAAAGCGT	520	
		TTCGGCTTGACTCGGCTGA		
	VEB-1 to VEB-6	CATTTCCCGATGCAAAGCGT	648	
		CGAAGTTTCTTTGGACTCTG		
Multiplex V GES and OXA-48-like	GES-1 to GES-9 and GES-11	AGTCGGCTAGACCGGAAAG	399	
		TTTGTCCGTGCTCAGGAT		
	OXA-48-like	GCTTGATCGCCCTCGATT	281	
		GATTTGCTCCGTGGCCGAAA		
Multiplex VI IMP, VIM, and KPC	IMP variants except IMP-9, IMP-16, IMP-18, IMP-22 and IMP-25	TTGACACTCCATTTACDG	139	
		GATYGAGAATTAAGCCACYCT		
	VIM variants, including VIM-1 and VIM-2	GATGGTGTTTGGTCGCATA	390	
		CGAATGCGCAGCACCAG		
	KPC-1 to KPC-5	CATTCAAGGGCTTTCTTGCTGC	538	
		ACGACGGCATAGTCATTTGC		
*mrp*H	Mannose-Resistant/Proteus-like fimbriae	F: CCTTGTTATGGTTGGCCTGT	444	[21]
		R: AGCCAGATGCGATAACCAAC		
*pmf*A	Major fimbrial subunit (PmfA)	F: CAAATTAATCTAGAACCACTC	618	[22]
		R: ATTATAGAGGATCCCTTGAAGGTA		
*uca*	Fimbriae	F: GCTGGCTCATCTATGGCGTA	453	[23]
		R: AGCGGTAGATTGTCCGGTTG		
*atf*A	A structural subunit (AtfA)	F: CATAATTTCTAGACCTGCCCTAGCA	382	[24]
		R: ATTATAGAGGATCCCTTGAAGGTA		
*pt*A	Proteases	F: CCACTGCGATTATCCGCTCT	686	[25]
		R: ATCGGCAGAAGTGACAAGCA		
*hly*A	Hemolysin	F: AACAAGGATAAGCACTGTTCTGGCT	1177	[26]
		R: ACCATATAAGCGGTCATTCCCGTCA		
*rsb*A	Histidine-containing phosphotransmitter	F: TTGAAGGACGCGATCAGACC	467	
		R: ACTCTGCTGTCCTGTGGGTA		
*lux*S	Autoinducer	F: GTATGTCTGCACCTGCGGTA	464	[27]
		R: TTTGAGTTTGTCTTCTGGTAGTGC		
*acr*A	Membrane fusion protein	F: TTGAAATTACGCTTCAGGAT	189	[28]
		R: CTTAGCCCTAACAGGATGTG		
*acr*B	Inner membrane transporter	F: CGTACACAGAAAGTGCTCAA	183	[28]
		R: CGCTTCAACTTTGTTTTCTT		
*rsm*A	Repressor of secondary metabolites	F: TAGCGAGTGTTGACGAGTGG	562	[22]
		R: AGCGAGGTGAAGAACGAGAA		
*hmp*A	Flavohemoprotein	F: CCAGTGAATTAACGGCAGGT	709	[22]
		R: CGTGCCCAGTAATGGCTAAT		
*mrp*A	MR/P fimbrial adhesion	F: TTTCAGGAAACAAAAGATG	648	[22]
		R: TTCTTACTGATAAGACATTG		
*zapD*	Outer membrane protein	F: GGGGTAAAACAACGGCATCA	217	This study
		R: ACATCAACCATCGTTCGCTG		
*zap*A	Metalloprotease	F: ACCGCAGGAAAACATATAGCCC	540	[22]
		R: GCGACTATCTTCCGCATAATCA		

**Table 2:** Distribution of *P. mirabilis* isolates recovered from patients across different age groups. Only statistically significant *p*-values are shown.

Age Groups	Male (%)	Female (%)	Total (%)	*p*-Value
<1	2 (4.8)	0	2 (1.9)	
1–10	3 (7.1)	8 (12.9)	11 (10.6)	
11–20	0	3 (4.8)	3 (2.9)	
21–30	2 (4.8)	5 (8.1)	7 (6.7)	
31–40	3 (7.1)	9 (14.5)	12 (11.5)	
41–50	5 (11.9)	5 (8.1)	10 (9.6)	
51–60	2 (4.8)	6 (9.7)	8 (7.7)	
61–70	6 (14.3)	10 (16.1)	16 (15.4)	
71–80	9 (21.4)	12 (19.4)	21 (20.2)	
81–90	8 (19.1)	3 (4.8)	11 (10.6)	
91–100	2 (4.8)	1 (1.6)	3 (2.9)	
Total	42 (40.4)	62 (59.6)	104	0.0071

**Table 3:** Association between biofilm-forming capacity and antimicrobial resistance among *P. mirabilis* isolates.

Antibiotics	Strong n = 53 n (%)	Moderate n = 47 n (%)	Weak n = 4 n (%)	Fisher’s Exact Test * p-Value **
Ampicillin	15 (28.3)	14 (25.53)	2 (50)	1
Piperacillin/tazobactam	0 (0)	0 (0)	0 (0)	NA
Ceftriaxone	6 (11.32)	10 (21.27)	1 (25)	0.319
Cefoxitin	0 (0)	0 (0)	0 (0)	NA
Imipenem	1 (1.89)	0 (0)	0 (0)	1
Meropenem	0 (0)	0 (0)	0 (0)	NA
Gentamicin	14 (26.41)	14 (25.53)	2 (50)	0.606
Amikacin	2 (3.77)	0 (0)	0 (0)	0.535
Kanamycin	22 (41.51)	20 (42.55)	1 (25)	0.886
Ciprofloxacin	13 (24.52)	10 (21.27)	2 (50)	0.481
Ofloxacin	10 (18.87)	13 (27.66)	1 (25)	0.543
Levofloxacin	13 (24.52)	12 (25.53)	1 (25)	1
Trimethoprim/Sulfamethoxazole	31 (58.49)	22 (46.81)	1 (25)	0.102
Amoxicillin/Clavulanic acid	2 (3.77)	1 (2.13)	0 (0)	1
Ceftazidime	10 (18.87)	3 (6.38)	1 (25)	0.192
Cefepime	6 (11.32)	3 (6.38)	0 (0)	1
Cefotaxime	9 (16.98)	11 (23.40)	2 (50)	0.293

* The association was analyzed by comparing biofilm formation across resistant and susceptible isolates for each antimicrobial agent. ** *p*-values less than 0.05 are considered statistically significant.

**Table 4:** Prevalence of virulence- and biofilm-associated genes among biofilm-producing *P. mirabilis* isolates.

Virulence Genes	Biofilm Formation				
	Strong n (%)	Moderate n (%)	Weak n (%)	Total n (%)	Fisher’s Exact Test (*p*-Value) **
*ure*C	53 (100)	47 (100)	4 (100)	104 (100)	NA *
*ure*R	53 (100)	47 (100)	4 (100)	104 (100)	NA
*rsm*A	48 (90.6)	46 (97.9)	4 (100)	98 (94.2)	0.111
*hmp*A	51 (96.2)	46 (97.9)	4 (100)	101 (97.1)	0.644
*mrp*A	49 (92.5)	42 (89.4)	4 (100)	95 (91.4)	0.814
*mrp*H	39 (73.6)	37 (78.7)	3 (75)	79 (76)	0.154
*pmf*A	52 (98.1)	44 (93.6)	4 (100)	100 (96.2)	0.307
*atf*A	50 (94.3)	46 (97.9)	4 (100)	100 (96.2)	1
*uca*	46 (86.8)	41 (87.2)	3 (75)	90 (86.5)	0.141
*pta*	50 (94.3)	45 (95.7)	4 (100)	99 (95.2)	1
*lux*S	53 (100)	47 (100)	4 (100)	104 (100)	NA
*rsb*A	53 (100)	46 (97.9)	4 (100)	103 (99)	0.49
*acr*A	53 (100)	47 (100)	4 (100)	104 (100)	NA
*acr*B	50 (94.34)	44 (93.62)	4 (100)	98 (94)	0.075
*hly*A	0	0	0	0	NA

* NA in Fisher’s Exact Test column indicates that the gene in question was expressed in 100% of samples across all biofilm formation groups (Strong, Moderate, and Weak). In such cases, there is no variation between groups for the test to assess statistical significance, rendering the value Not Applicable. ** *p*-values less than 0.05 are considered statistically significant.

**Table 5:** Relationship between ERIC-PCR clonal clusters and biofilm-forming capacity of *P. mirabilis* isolates.

Biofilm Formation	Group 1		Group 2		Group 3	
	Cluster A (%)	Cluster B (%)	Cluster C (%)	Cluster D (%)	Cluster E (%)	Cluster F (%)
Strong	19 (73.1)	10 (47.6)	7 (46.7)	10 (38.5)	6 (75)	1 (12.5)
Moderate	6 (23.1)	10 (47.6)	7 (46.7)	16 (61.5)	2 (25)	6 (75)
Weak	1 (3.9)	1 (4.8)	1 (6.7)	0	0	1 (12.5)

**Table 6:** Distribution of antimicrobial resistance profiles across ERIC-PCR clonal clusters.

	Group 1		Group 2		Group 3	
Antibiotics	Cluster A (%)	Cluster B (%)	Cluster C (%)	Cluster D (%)	Cluster E (%)	Cluster F (%)
Ampicillin	7 (26.9)	4 (19.1)	5 (33)	12 (46.2)	2 (25)	1 (12.5)
Amikacin	1 (3.9)	0 (0)	1 (6.7)	0 (0)	0 (0)	0 (0)
Imipenem	0	0	1 (6.7)	0 (0)	0 (0)	0 (0)
Kanamycin	11 (42.3)	6 (28.6)	8 (53.3)	13 (50)	4 (50)	2 (25)
Cefoxitin	0 (0)	0 (0)	0 (0)	0 (0)	0 (0)	0 (0)
Trimethoprim/ sulfamethoxazole	12 (46.2)	11 (52.4)	9 (60)	12 (46.2)	6 (75)	2 (25)
Ciprofloxacin	7 (26.9)	3 (14.3)	6 (40)	5 (19.2)	3 (37.5)	2 (25)
Piperacillin/ tazobactam	0 (0)	0 (0)	0 (0)	0 (0)	0 (0)	0 (0)
Gentamicin	8 (30.8)	7 (33.3)	4 (26.7)	9 (34.6)	2 (25)	0 (0)
Ceftriaxone	2 (7.69)	3 (14.3)	4 (26.7)	6 (23.1)	1 (12.5)	1 (12.5)
Levofloxacin	8 (30.8)	3 (14.3)	6 (40)	7 (26.9)	2 (25)	2 (25)
Ofloxacin	8 (30.8)	3 (14.3)	3 (20)	8 (30.8)	1 (12.5)	1 (12.5)
Meropenem	0 (0)	0 (0)	0 (0)	0 (0)	0 (0)	0 (0)
Cefotaxime	6 (23.1)	4 (19.1)	3 (20)	7 (26.9)	1 (12.5)	2 (25)
Cefepime	3 (11.5)	1 (4.8)	2 (13.3)	2 (7.7)	1 (12.5)	0 (0)
Amoxicillin/ clavulanate	1 (3.9)	0 (0)	1 (6.7)	1 (3.9)	0 (0)	0 (0)
Ceftazidime	5 (19.2)	2 (9.5)	3 (20)	2 (7.7)	1 (12.5)	1 (12.5)

**Table 7:** Distribution of virulence-associated genes across ERIC-PCR clonal clusters of *P. mirabilis* isolates.

Virulence Genes	Group 1		Group 2		Group 3	
	Cluster A (%)	Cluster B (%)	Cluster C (%)	Cluster D (%)	Cluster E (%)	Cluster F (%)
*zap*A	26 (100)	21 (100)	15 (100)	25 (96.2)	8 (100)	8 (100)
*zap*D	25 (96.2)	21 (100)	15 (100)	26 (100)	8 (100)	8 (100)
*rsb*A	26 (100)	20 (95.2)	15 (100)	26 (100)	8 (100)	8 (100)
*hmp*A	26 (100)	19 (90.5)	15 (100)	25 (96.2)	8 (100)	8 (100)
*atf*A	25 (96.2)	19 (90.5)	15 (100)	25 (96.2)	8 (100)	8 (100)
*pta*	25 (96.2)	18 (85.7)	15 (100)	26 (100)	7 (87.5)	8 (100)
*acr*B	26 (100)	21 (100)	14 (93.3)	22 (84.6)	8 (100)	7 (87.5)
*rsm*A	25 (96.2)	19 (90.5)	14 (93.3)	24 (92.3)	8 (100)	8 (100)
*pmf*A	25 (96.2)	21 (100)	13 (86.7)	23 (88.5)	8 (100)	7 (87.5)
*mrp*A	24 (92.3)	18 (85.7)	15 (100)	23 (88.5)	8 (100)	7 (87.5)
*uca*	24 (92.3)	17 (81)	12 (80)	22 (84.6)	8 (100)	7 (87.5)
*mrp*H	20 (76.9)	16 (76.2)	10 (66.7)	20 (76.9)	6 (75)	6 (75)

## Data Availability

The original contributions presented in this study are included in the article/Supplementary Material. Further inquiries can be directed to the corresponding author.

## Supplementary Materials

The following supporting information can be downloaded at https://www.mdpi.com/article/10.3390/microorganisms14061242/s1. Table S1: PCR temperature program.

## Abbreviations

UTI	Urinary Tract Infection
AMR	Antimicrobial Resistance
MDR	Multidrug-Resistant
MLST	Multilocus Sequence Typing
ESBL	Extended-Spectrum β-Lactamase
ESCR	Extended-Spectrum Cephalosporin Resistance
CA-UTI	Catheter-Associated UTIs
WGS	Whole-Genome Sequencing
PCR	Polymerase Chain Reaction
ERIC-PCR	Enterobacterial Repetitive Intergenic Consensus PCR
LB	Luria–Bertani
CFU	Colony-Forming Unit
OD	Optical Density
ODc	Optical density cut-off value
PBS	Phosphate-Buffered Saline
TSB	Tryptic Soy Broth
CLSI	Clinical and Laboratory Standards Institute
ATCC	American Type Culture Collection
MHA	Mueller–Hinton Agar
DNA	Deoxyribonucleic Acid
*ure*C	Urease C gene
*ure*R	Urease Regulator gene
*rsm*A	Regulatory small RNA gene
*hmp*A	Hemophore-like protein gene
*mrp*A	Mating-pair formation protein A gene
*mrp*H	Mating-pair formation protein H gene
*pmf*A	*Proteus mirabilis* fimbriae gene
*atf*A	Adhesion and invasion protein gene
*uca*	Uropathogenic-associated adhesin gene
*pta*	Phosphotransferase gene
*lux*S	Quorum sensing gen
*rsb*A	Response regulator gene
*zap*A	ZAP-associated protein gene
*zap*D	ZAP-associated protein gene
*acr*A	Acridine resistance gene
*acr*B	Acridine resistance gene
*hly*A	Hemolysin gene
*bla*TEM	Gene encoding TEM β-lactamase
*bla*CTX-M-2	Gene encoding CTX-M-2 β-lactamase
*bla*KPC	Gene encoding KPC β-lactamase
*bla*CTX-M-9	Gene encoding CTX-M-9 β-lactamas
*bla*OXA-1-like	Gene encoding OXA-1-like β-lactamase
*bla*SHV	Gene encoding SHV β-lactamase
*bla*GES	Gene encoding GES β-lactamase
*bla*PER	Gene encoding PER β-lactamase
