# Supplementary material for: Biofilm Formation, Virulence-Associated Genes, and Antimicrobial Resistance in Proteus mirabilis Isolates from Urinary Tract Infections in Iran
Source: Microorganisms. 2026 May 31;14(6):1242. doi: 10.3390/microorganisms14061242 (PMC13304133; doi:10.3390/microorganisms14061242)
Supplement: Supplementary file 1 [file microorganisms-14-01242-s001.zip › microorganisms-4191529-supplementary.pdf]

Table S1. PCR temperature program

Gene	Cycles	Initial Denaturation	Denaturation	Annealing	Extension	Final Extension
*zap*A	35	95 °C, 5 min	95 °C, 1 min	53 °C, 1 min	72 °C, 10 min	72 °C, 10 min
*rsm*A	35	94 °C, 5 min	94 °C, 1 min	56 °C, 1 min	72 °C, 10 min	72 °C, 10 min
*hmp*A	35	94 °C, 5 min	94 °C, 1 min	57 °C, 45 sec	72 °C, 1 min	72 °C, 10 min
*mrp*A	30	94 °C, 3 min	94 °C, 30 sec	50 °C, 30 sec	72 °C, 30 sec	72 °C, 7 min
*ure*C	35	95 °C, 5 min	95 °C, 1 min	52 °C, 1 min	72 °C, 5 min	72 °C, 10 min
*ure*R	30	94 °C, 4 min	94 °C, 40 sec	50 °C, 1 min	72 °C, 20 sec	72 °C, 10 min
*zap*D	30	94 °C, 5 min	94 °C, 1 min	56 °C, 1 min	72 °C, 10 min	72 °C, 10 min
